# The prevalence of dental anxiety and its association with pain and other variables among adult patients with irreversible pulpitis

**DOI:** 10.1186/s12903-018-0563-x

**Published:** 2018-06-07

**Authors:** Lei Dou, Margaret Maria Vanschaayk, Yan Zhang, Xiaoming Fu, Ping Ji, Deqin Yang

**Affiliations:** 1 Stomatological Hospital of Chongqing Medical University, 426 Song Shi Bei Road, Chongqing, 401147 China; 2 Chongqing Key Laboratory of Oral Diseases and Biomedical Sciences, 426 Song Shi Bei Road, Chongqing, 401147 China; 3 Chongqing Municipal Key Laboratory of Oral Biomedical Engineering of Higher Education, 426 Song Shi Bei Road, Chongqing, 401147 China; 4 Wake Forest Institute for Regenerative Medicine, 391 Technology Way NE, Winston-Salem, NC 27101 USA

**Keywords:** Adult people, Associated factors, Dental anxiety, Irreversible pulpitis, Pain

## Abstract

**Background:**

The aim is to investigate the prevalence of dental anxiety and its association with pain and other related factors in adult patients with irreversible pulpitis.

**Methods:**

One hundred and thirty patients with irreversible pulpitis were included in this cross-sectional study. Participants were asked to fill out an information table and a battery of questionnaires to assess their level of dental anxiety, pain at their first and most recent dental experience, and pain intensity before/during the present endodontic treatment. The level of anxiety that participants displayed during the present treatment was also evaluated by the dentists using an anxiety rating scale. Data were analyzed by t-test, ANOVA, and Spearman correlation tests.

**Results:**

83.1% of participants suffered from moderate or high dental anxiety, and 16.2% met criteria for specific phobia. Subjects who had higher MDAS scores were more likely to postpone their dental visits (*P* < 0.05). Subjects who had bad experiences at their most recent dental visit were more anxious (*P* < 0.05). Pain at the most recent dental visit (*P* < 0.01) or before the present dental visit (*P* < 0.05) was important factor correlating with dental anxiety among participants. Notably, 36.2% of participants displayed moderate or severe anxiety during this present visit for endodontic treatment based on dentist’s judgement.

**Conclusions:**

A high percentage of people with irreversible pulpitis suffer from dental anxiety. Pain at the most recent dental visit and during endodontic treatment have strongly positive association with dental anxiety. Effective pain control in endodontics is beneficial to manage the anxiety.

## Background

Dental anxiety involves the nervousness of dentistry and of receiving dental care, which commonly causes poor oral health, evasion of dental treatment, and may sometimes lead to worsening of oral health-related quality of life [1]. A high proportion of adults experience some levels of dental anxiety, ranging from mild to severe dental anxieties. It is estimated that about 3–16% of adults suffer from dental phobia [2–6]. A number of factors might play a part in the development of dental anxiety. They include direct conditioning through negative dental experiences, indirect learning through other people’s or various media’s influence, pain, personality characteristics, and environmental factors [1, 6, 7]. Moreover, becoming worried over the possibility of experiencing pain during dental visits might also significantly contribute to the development of dental anxiety [8, 9].

Irreversible pulpitis is an infuriating medical condition that involves spontaneous and impulsive pain. Such pain might compel the patients suffering from pulpitis to seek emergency dental care. Nonetheless, the fear of pain during and after treatment might prompt some level of dental anxiety in patients, leading to delay or avoidance of treatment.

The typical treatment for irreversible pulpitis is endodontic therapy, which is alleged to be physically and psychologically distressing for patients. The endodontic therapy process includes injection, drilling, extirpation of pulp, root canal shaping, cleaning, and obturation, which may provoke dental fear in patients. The success rate of achieving deep pulpal anaesthesia lessens in patients with irreversible pulpitis. It is broadly acceptable that achieving anaesthesia in teeth with irreversible pulpitis is more complex in comparison with teeth with normal, healthy pulps [10]. Inflammatory transformations may cause high rates of anaesthesia failure of teeth affected by irreversible pulpitis [11]. Even with local anaesthesia, a portion of patients would still feel pain when obtaining endodontic therapy, and experience stressful post-operative pain [10]. A distressing dental experience might lead people to become fearful and anxious, which may also lead to fear of future dental visits. Furthermore, dental anxiety might easily develop among patients with negative dental experiences since such patients may postpone dental visits or fail to obey dentists’ instructions. Identifying dental anxiety among patients can make it possible for dentists to predict patient behaviour and be better prepared with measures that will help the patient lessen their anxiety. Since irreversible pulpitis is a dental emergency, patients with irreversible pulpitis who seek dental care are a representative of the entire population with a high predominance of dental anxiety. It was expected that individuals with high dental anxiety would report more pain during endodontic treatment than individuals with low levels of dental anxiety. Nevertheless, few literatures available could presented the prevalence of dental anxiety and related factors correlating with dental anxiety among Chinese adult patients with irreversible pulpitis seeking emergency dental service.

Therefore, this study intends to evaluate the prevalence of dental anxiety and its relation with pain, among other variables, in Chinese adult patients with irreversible pulpitis seeking dental care.

## Methods

One hundred and thirty Chinese adult patients in the Stomatological Hospital of Chongqing Medical University were included in this study according to inclusion criteria. The inclusion criteria were: 1) older than 18 yrs. and younger than 70 yrs., 2) diagnosed with irreversible pulpitis by three designated qualified dentists who participated in this study, 3) with pulp inflammation caused by dental caries. Those who were suffering from psychiatric diseases, taking any analgesics/antipsychotic drug within 24 h or completely illiterate were excluded from the study. A written informed consent was obtained from each participant. If a patient could not read and sign the consent form, we defined him as illiterate and did not include him in the study. Approval for the study was obtained from the institutional ethics committee of Chongqing medical university.

In this cross-sectional study, each participant was provided with a battery of questionnaires including three sections. The first section was presented in an information table, containing age, gender, educational background, employment status, self-perceived oral `health status, history of dental visit as well as endodontic therapy. The educational background was divided into “uneducated”, “high school”, “degree/diploma”, and “post graduate”. The employment status was categorized into “employed (full-time)”, “employed (part-time)”, “unemployed”, “student”, and “retired”. History of dental visit was further divided into “dental visits experience”, “postponement of dental visit”, and “perception of first and most recent dental visits”. Besides, history of endodontic therapy had “endodontic therapy experience”, “perception of endodontic experience” and “indirect endodontic therapy experience from others or various media”.

The second section was used in assessing the levels of dental anxiety among participants. This section was contained the Chinese version of Modified Dental Anxiety Scale [12] (MDAS), divided into a 5-item measure to assess fear of dental procedures, including before going for treatment, waiting for treatment, drilling, cleaning, and local anesthetic injections. Ratings of 1–5 indicated “not anxious”, “slightly anxious”, “fairly anxious”, “very anxious” and “extremely anxious”, correspondingly. A clinical anxiety rating scale known as CARS was also used during the endodontic treatment. The investigator is instructed to evaluate the extent of anxiety which the patients display during endodontic treatment on the basis of patients’ expressions, talks and behaviors. Ratings of 1–5 in CARS indicate “no”, “mild”, “moderate”, “severe” and “very severe” respectively in relation to prevalence of feelings in the patients [13]. Dental phobia can be described when total MDAS score was 19 or above. The investigators were trained and calibrated prior to this study, and the intra-examiner reliability was evaluated using the kappa test. A kappa value of 0.850 was achieved.

The third section was about a Visual Analogue Scale (VAS) in evaluating the pain intensity for first and most recent dental experiences, together with the pain before and during the present endodontic therapy. This pain scale was devised to be an 11-point scale ranging from 0 (no pain) to 10 (worst possible pain) as a way to measure physical pain intensity.

Mean total MDAS score and CARS score were calculated for all the categorized variables. Independent sample *t*-test and one-way analysis of variance (ANOVA) was done to compare mean MDAS score and CARS score between categories in a same variable. For example, to compare mean MDAS and CARS scores between category of gender, scores were compared between males and females. Tukey’s Post Hoc Test was done to control for multiple comparisons. Spearman rank correlation was done to assess the strength of association between MDAS and pain at the first/most recent dental experience or pain before/during this endodontic treatment. Pearson and Spearman rank correlation was used to evaluate the association between CARS score during endodontic treatment and the variables (pain at the first/most recent dental experience or pain before/during this present endodontic treatment, Total MDAS, Q1, Q2, Q3, Q4, Q5 of MDAS).

## Results

Among the 130 respondents, 46.9% were males and 53.1% were females. Mean age of the participants was 41.8 years (SD =14.25). Mean total score for dental anxiety on MDAS was 14.17 (SD = 4.76). Cronbach’s alpha was 0.91 in the current sample. Based on the MDAS score, 16.9% of the subjects were identified to be less anxious (5–9 total score), 66.9% were moderately anxious (10–18 total score), and 16.2% (≥19 total score) were seriously anxious (defined as dental phobia). Table 1 show mean MDAS score for all the categorized variables and difference among groups. Subjects who had higher MDAS scores were more likely to postpone their dental visit (*P* < 0.05). No correlation was found between MDAS and variables including age, gender, educational background, employment status, self-perceived oral health status. Negative dental experience during treatment demonstrated the strong relation with dental anxiety. Participants who had bad experiences at their most recent dental visit were more anxious compared to those with good experiences (*P* < 0.05). Pain at most recent dental visit or before the present dental visit was the important factor correlating with dental anxiety among participants (Table 2). The mean CARS score was 2.24 ± 0.96. Notably, 36.2% participants displayed moderate or severe anxiety according to their expressions, behaviors or talks during this present visit for endodontic treatment. A positive correlation was also observed between MDAS and CARS (*P* < 0.001). Subjects who experienced more pain at the most recent dental visit were more likely to display anxiety when they received endodontic treatment (Table 3). Participants show higher CARS score if their past endodontic experiences were bad (*P* < 0.01). Meanwhile, for participants who didn’t receive endodontic therapy before, negative attitude from others’ experience or other media were also associated with more anxiety during this present endodontic therapy (*P* < 0.001).

**Table 1:** Variables assessed in the study with sample size, percentage, mean total MDAS score and statistical test

Variables		Number	Pecentage	Mean total Score	Statistical test	*P*-value
Gender	Male	61	46.9%	14.08 ± 4.46	t test	> 0.05
	Female	69	53.1%	14.25 ± 5.04		
Age	18-30 years	29	22.3%	13.90 ± 4.59	ANOVA F = 0.943	> 0.05
	30-45 years	52	40.0%	14.71 ± 5.04		
	46-60yearrs	23	17.7%	14.70 ± 5.08		
	60-70 yrs	26	20.0%	12.92 ± 4.06		
Educational level	Uneducated	26	20.0%	14.92 ± 5.08	ANOVA F = 0.648	> 0.05
	High school	49	37.7%	14.47 ± 4.55		
	Degree/Diploma	38	29.2%	13.37 ± 4.87		
	Post graduation	17	13.1%	13.94 ± 4.76		
Employment	Employed (full-time)	72	55.4%	13.93 ± 5.18	ANOVA F = 0.490	> 0.05
	Employed (Part-time)	8	6.2%	14.87 ± 4.97		
	unemployed	12	9.2%	15.75 ± 4.18		
	Student	9	6.9%	14.67 ± 2.18		
	Retired	29	22.3%	13.76 ± 4.51		
Self-perceived oral health	Good	30	23.1%	13.57 ± 3.86	ANOVA F = 1.163	> 0.05
	Average	54	41.5%	13.78 ± 4.82		
	Poor	46	35.4%	15.02 ± 5.19		
Dental experience	Yes	85	65.4%	14.51 ± 4.96	t test	> 0.05
	No	45	34.6%	13.53 ± 4.34		
First dental visit	< 12 years old	20	23.5%	16.85 ± 4.78	ANOVA F = 2.390	> 0.05
	12–18 years old	27	31.8%	14.15 ± 5.25		
	> 18 years old	30	35.3%	13.17 ± 4.52		
	unclear	8	9.4%	14.88 ± 4.73		
First dental experience	Good	19	22.4%	14.89 ± 4.93	ANOVA F = 1.060	> 0.05
	Not bad	42	49.4%	13.77 ± 4.69		
	Bad	24	28.2%	15.57 ± 5.46		
Frequency of dental visits	Every 3 months	12	14.1%	13.67 ± 3.87	ANOVA F = 0.157	> 0.05
	Every 6 months	17	20.0%	14.35. ± 5.72		
	Every 12 months	30	3.35%	14.67 ± 5.27		
	Less frequently	26	30.6%	14.81 ± 4.74		
Most recent dental experience	Good	22	25.9%	12.09 ± 3.96	ANOVA F = 4.122	< 0.05*
	Not bad	31	36.5%	14.84 ± 5.05		
	Bad	32	37.6%	15.84 ± 5.04		
The length of time since most recent dental visit	Within 3 months	19	22.4%	14.95 ± 5.78	ANOVA F = 0.108	> 0.05
	3–12 months	36	42.4%	14.47 ± 5.01		
	Longer than 12 months	30	35.3%	14.27 ± 4.49		
Postponement of dental visit	Yes	69	53.1%	15.00 ± 4.72	t test	< 0.05
	No	61	46.9%	13.23 ± 4.67		
Endodontic therapy experience	Yes	39	30.0%	14.69 ± 4.69	t test	> 0.05
	No	91	70.0%	13.95 ± 4.80		
Perception of endodontic therapy	Good	9	23.1%	12.67 ± 3.87	ANOVA F = 2.985	> 0.05
	Slightly bad	12	30.8%	13.42 ± 3.53		
	Very bad	18	46.2%	16.56 ± 5.20		
Indirect endodontic experience	Positive	16	17.6%	13.31 ± 5.07	ANOVA F = 2.985	> 0.05
	Neutral	27	29.7%	14.37 ± 4.00		
	Negative	35	38.5%	14.63 ± 5.24		
	Not hear of this therapy	13	14.3%	12.00 ± 4.66		

*There was a significant difference between the “good” and “bad” groups

**Table 2:** The correlation between total MDAS Score and variables in the study

Variables	Spearman Correlation	*P*-value
Pain at the most recent dental visit	0.283	0.009**
Pain at the firstdental visit	0.180	0.100
Pain before the present visit	0.191	0.030*
Pain during the present visit	0.431	0.000***
Clinical anxiety rating scale by dentist(CARS)	0.308	0.000***

*P < 0.05

**P < 0.01

***P < 0.001

**Table 3:** The correlation between CARS score and variables in the study

Variables	Spearman Correlation	*P*-value
Pain at the first dental visit	0.167	0.126
Pain at the most recent dental visit	0.269	0.013*
Pain before the present visit	−0.137	0.119
Pain during the present visit	0.121	0.169
Q1 of MDAS (If you went to your Dentist for Treatment tomorrow)	0.289	0.001**
Q2 of MDAS (If you were sitting in the waiting room/waiting for treatment)	0.284	0.001**
Q3 of MDAS (If you were about to have a tooth drilled)	0.280	0.001**
Q4 of MDAS (If you were about to have your teeth scaled and polished)	0.307	0.000***
Q5 of MDAS (If you were about to have a local anaesthetic injection in your gum)	0.242	0.006**

*P < 0.05

**P < 0.01

***P < 0.001

## Discussion

Findings of the present study suggest that there is a higher prevalence of dental anxiety among patients with irreversible pulpitis and are seeking emergency dental care as compared with the results in previous studies [6, 8, 14]. In this current study, 83.1% of subjects reported moderate to high levels of dental anxiety, which is higher than the findings in previous studies. A prior study by Tellez [8] pointed out that only half (49.2%) subjects suffered from moderate to high anxiety. In the meantime, 54.8 and 58.8%, respectively, were indicated in two other studies [6, 14]. The prevalence rates of dental phobia also differ in various studies. 16.2% of the participants included met the criteria of dental phobia (MDAS score ≥ 19), which is relatively higher than a lot of other previous studies [6, 15, 16]. Appukuttan et al., [6] reported that 3% of people suffered from dental phobia in an India population. Moreover, Halonen [15] also reported that 11.3% met the criteria of dental phobia in Finland, while Humphris [16] reported that in Britain, it was 11.6%. The constitution of integrated subjects in these researches contributes to the difference. In these studies, all participants, including regular and emergency patients, were also included. However, it is notable that patients were recruited from a dental emergency-seeking population in this current study. It is assumed that all the participants seek emergency dental care due to of acute pain provoked by irreversible pulpitis. All patients suffered from moderate to severe pain previous to this survey questionnaire. The pain before treatment may be a factor that is associated with the extent of anxiety among patients. This was proved by the results of the present study. Pain before endodontic treatment was realized to have a significant correlation with the mean MDAS scores (*P* < 0.05). Likewise, endodontic therapy is a traumatic procedure accompanied by pain, particularly without effective pain-managing modality. Some people who have experienced endodontic treatment report that it is an uncomfortable experience [17]. The aforementioned may increase the level of dental anxiety. It is supposed that patients with irreversible pulpitis were more anxious compared with the general population or other patients seeking dental care. The findings of a previous study also demonstrated that a higher number of emergency patients met the criteria for the diagnosis of dental phobia than regular patients (35.7% versus 14.1%) [8].

Irreversible pulpitis is an irritating disease. Severe and impulsive pain necessitated patients to go to dental emergency for treatment. Patients seeking to relieve pain were often afraid of the pain provoked during the examination and the treatment procedure. A series of procedure, including local anesthetic injection, drilling tooth and extirpation of dental pulp are invasive [18]. This could be anxiety-provoking stimuli. Even though local anaesthesia is broadly applied in the endodontic treatment, an entirely effective control of pain is still difficult [19]. This means that a portion of patients would still experience a distressing pain when they receive endodontic treatment. Pain plays a crucial role in the development of dental anxiety. Studies have shown that the fear of pain during dental treatment is the elemental reason for dental phobia [11]. In the current study, it was established that patients who were dentally anxious would feel more pain during endodontic treatment (*P* < 0.001). For effectual management of dentally anxious patients, particularly these dentally phobic individual, more effectual local anaesthesia and anti-anxiety strategies ought to be enhanced in endodontic treatment in future. In addition, sufficient analgesics are also required in controlling postoperative pain.

There is a shared relationship between dental anxiety and pain. In the current study, patients with prior negative experiences tend to have higher dental anxiety. Pain at the most recent dental visit considerably envisaged higher anxiety scores. In the meantime, dentally anxious patients felt more pain in the present traumatic dental visits as compared with non-anxious patients. Subsequently, anxiety of these dentally anxious patients would worsen.

In this study, a positive correlation was identified between MDAS and CARS (*P* < 0.001). Patients who had a higher MDAS scores depicted higher anxiety during the present endodontic therapy through their behaviours, expressions, and talks. This implied that MDAS is an efficient predictor of anxiety that patients subsequently portray in the ensuing endodontic therapy. Thus, performing a simple questionnaire (MDAS) is suggested before patients receive endodontic therapy. The score of MDAS can permit the dentist to find anxious individuals, anticipate patient behaviours, and therefore help ease the patient’s anxiety in the subsequent endodontic therapy procedures.

Indirect negative experiences can also play a role in dental anxiety. In this current study, participants with bad indirect experiences of endodontic treatment might portray higher anxiety during the present endodontic therapy. Even though they did not have a bad experience with endodontic treatment prior, their dental fear toward endodontic treatment might build up depending on what they hear from others’ bad experiences or negative observations of dentistry. The negative display of dentistry or endodontic in mass media and cartoons may also lead to the advancement of dental fear [6, 7]. Lack of insightful comprehension of the whole issue might also make some people feel extremely anxious before treatment [6]. Therefore, dentists should make more efforts in alleviating anxiety of patients and offer a good dental experience to them. Moreover, a good environment needs to be established, wherein people can obtain adequate knowledge of endodontic therapy. Positive view of dentistry should also be shown through various media to form a good image of endodontic therapy to patients.

This analytical cross-sectional study was carried out to investigate the association among dental anxiety, pain, and other variables in Chinese adult patients with irreversible pulpitis. The results of this study disclosed the correlation among dental anxiety and some varieties including bad experience during treatments and pain during or before the dental visit, which could help provide information in anxiety management. However, this study has the limitation in making a valid conclusion about the causality among dental anxiety, pain as well as bad experiences in dental visits owing to design type. Further prospective controlled trials are needed to confirm the causality between dental anxiety and pain management in adult patients with irreversible pulpitis seeking dental care. Improved level of pain-controlling measure (general anesthesia, etc.) could be taken on some extremely-anxious patients to investigate the effect of enhanced pain management on dental anxiety.

## Conclusions

Based on the results of this study, dental anxiety is found to be widespread among patients with irreversible pulpitis. Dental anxiety among patients with irreversible pulpitis have a positive correlation with pain that they experienced. Effective pain-controlling strategies in endodontics may improve the management of dentally anxious patients.

## Abbreviations

CARS: Clinical anxiety rating scale
MDAS: Modified dental anxiety scale
Q1, Q2, Q3, Q4, Q5: Question 1–5 of modified dental anxiety scale
SD: Standard deviation
VAS: Visual analogue scale
